# Scaffold Morphing and In Silico Design of Potential BACE-1 (β-Secretase) Inhibitors: A Hope for a Newer Dawn in Anti-Alzheimer Therapeutics

**DOI:** 10.3390/molecules28166032

**Published:** 2023-08-12

**Authors:** Shiveena Bhatia, Manjinder Singh, Pratibha Sharma, Somdutt Mujwar, Varinder Singh, Krishna Kumar Mishra, Thakur Gurjeet Singh, Tanveer Singh, Sheikh Fayaz Ahmad

**Affiliations:** 1 Chitkara College of Pharmacy, Chitkara University, Rajpura 140401, Punjab, Indiapratibhasharma2606@gmail.com (P.S.); somduttmujwar@gmail.com (S.M.); gurjeet.singh@chitkara.edu.in (T.G.S.); 2 Department of Pharmaceutical Sciences and Technology, Maharaja Ranjit Singh Punjab Technical University, Bathinda 151001, Punjab, India; varinderjassal17@gmail.com; 3 Chitkara University Institute of Engineering and Technology, Chitkara University, Rajpura 140401, Punjab, India; krishna.mishra@chitkara.edu.in; 4 Department of Neuroscience and Experimental Therapeutics, College of Medicine, Texas A&M Health Science Center, College Station, TX 77807, USA; 5 Department of Pharmacology and Toxicology, College of Pharmacy, King Saud University, Riyadh 11451, Saudi Arabia; fashaikh@ksu.edu.sa

**Keywords:** Alzheimer’s disease, β-secretase, β-amyloid, BACE-1, dementia, elenbecestat

## Abstract

Alzheimer’s disease (AD) is the prime cause of 65–80% of dementia cases and is caused by plaque and tangle deposition in the brain neurons leading to brain cell degeneration. β-secretase (BACE-1) is a key enzyme responsible for depositing extracellular plaques made of β-amyloid protein. Therefore, efforts are being applied to develop novel BACE-1 enzyme inhibitors to halt plaque build-up. In our study, we analyzed some Elenbecestat analogues (a BACE-1 inhibitor currently in clinical trials) using a structure-based drug design and scaffold morphing approach to achieve a superior therapeutic profile, followed by in silico studies, including molecular docking and pharmacokinetics methodologies. Among all the designed compounds, SB306 and SB12 showed good interactions with the catalytic dyad motifs (Asp228 and Asp32) of the BACE-1 enzyme with drug-likeliness properties and a high degree of thermodynamic stability confirmed by the molecular dynamic and stability of the simulated system indicating the inhibitory nature of the SB306 and SB12 on BACE 1.

## 1. Introduction

According to the World Health Organization, they estimate that in 2022, Alzheimer’s disease and associated dementias will be the seventh major cause of death globally. It is currently the paramount reason for the suffering of about 50 million patients (https://www.who.int/ (accessed on 6 June 2023)). The estimates point towards the probable increase in cases to around 13.8 million by 2060 [1,2]. The disease is disastrous in terms of mortality and morbidity and is characterized by deficits in the cognitive and motor abilities of the patients and dementia. It results due to neuron degeneration in the cortical and hippocampal regions of the brain, which control learning, memory, and cognitive abilities [3]. Alzheimer’s disease (AD) is associated with the formation of senile plaques and neurofibrillary tangles in the brain, which causes dementia and memory loss [4,5,6]. The primary hallmark of Alzheimer’s disease is the accumulation of β-amyloid proteins in the synapses of the cortical neurons and the formation of tau tangles inside the neurons due to the twisting of tau protein fibers. These accumulations lead to the neurons’ death by hampering the transfer of signals among the neurons [7,8]. The accumulation of β-amyloid proteins between the synaptic spaces results from the action of the BACE-1 (β-site amyloid precursor protein cleaving enzyme 1) enzyme. The enzyme catalyzes the reaction’s rate-determining step, producing toxic Aβ by cleaving the extracellular domain of the amyloid precursor protein (APP) [9]. It generates Aβ under stressful conditions triggering glial activation, the sequential proteolysis of type 1 membrane protein APP. The process of formation of Aβ from APP comprises the first step of cleavage of APP by the BACE-1 enzyme leading to the generation of a C99 fragment (a membrane-bound C-terminal) [10], followed by the splitting of C99, leading to the liberation of Aβ by γ-secretase enzyme comprised four transmembrane proteins (presenilin, nicastrin, Pen2, and Aph1). Another enzyme, α-secretase, can also cleave APP at a position within Aβ, blocking the formation of Aβ [10,11]. Since the BACE-1 enzyme is involved in the first step, the rate-determining step in producing Aβ, it is viewed as a viable target for anti-Alzheimer’s treatments. No BACE-1 inhibitor is commercially available for anti-AD treatment, but many of these have entered human clinical trials [12,13].

The BACE-1 enzyme is a 21 amino acid long peptide with a NH_2_ terminal followed by a pro-protein of 22–45 amino acid residues [14]. The catalytic domain of the BACE-1 enzyme comprises 46–460 amino acids residue. The enzyme’s active/catalytic site, known as the catalytic dyad, has two aspartic acid remains (Asp32 and Asp228), responsible for the catalytic action and form the prime interactive sites for all the potential BACE-1 inhibitors [15]. Other important residues comprising the active binding site of the BACE-1 enzyme are Thr329, Gly34, Arg235, Tyr71, Lys224, Trp115, Arg128, Gly230, Val332, Thr231, and Thr232. Targeting the catalytic dyad residues has been a major focus for designing the drug targeting BACE-1 inhibition [15,16].

It has been hypothesized that existing drugs can quickly be an alternative source for developing newer therapies by exploring other therapeutic roles. In this study, we screened the small molecular BACE-1 inhibitors in clinical trials and chose one of these molecules, elenbecestat, which is a small molecule inhibitor [N-[3-[(4a*S*, 5*R*, 7a*S*)-2-Amino-5-methyl-4,4a, 5,7-tetrahydrofuran [3,4-d][1,3]thiazin-7a-yl]-4-fluorophenyl]-5-(difluoromethyl)pyrazine-2-carboxamide] currently undergoing phase 3 clinical trials with significant efficacy in reducing Aβ levels in plasma and (cerebrospinal fluid) CSF. The subjects administered elenbecestat demonstrated fewer declines in functional cognition. But, the treatment also showed side effects like contact dermatitis, respiratory tract infections, nightmares and abnormal dreams, headaches, falls, diarrhea, etc. In the current study, we report some analogues of elenbecestat, designed using scaffold morphing and (structure-based drug design) SBDD approaches.

Scaffold morphing is a distinctive medicinal chemistry tool used for the lucid design of drugs via gradual modification in the parent compound to develop varied, novel molecules with better therapeutic potential [17]. The scaffold morphing techniques were applied to generate various structural analogues of elenbecestat with a good synthetic accessibility index, and then these were subjected to in silico pharmacokinetic studies to determine their drug-likeliness properties along with their potential to penetrate the (blood–brain barrier) BBB. After the molecular docking, on the basis of their binding interaction with BACE-1, two potential and promising candidates were found for future drug development into BACE-1 inhibitors. Finally, to validate the docking studies, molecular dynamic (MD) simulations were also performed to explore the interactions of the target-ligand complex in dynamic motion to evaluate the thermodynamic firmness and persuaded conformational changes at the BACE-1 gorge.

## 2. Results and Discussion

### 2.1. Scaffold Morphing through Bioisosteric Replacement

The chemical structure of elenbecestat was analyzed, and keeping the nucleus of the molecule pyrazine-2-carboxamide constant, multiple sites were switched using their bioisosteres by using the MolOpt web server (Table 1) [18]. The molecule elenbecestat was drawn in the software search bar, and analogues were developed using the four inbuilt protocols in the software. The analogues possessing improved pharmacokinetic, physicochemical, and pharmacodynamic properties were developed and sorted in the increasing order of their synthetic accessibility. After sorting out, 2003 molecules were found to possess a score ≤ 4.5; after further eliminating analogues with similar structures and properties, 1880 proceeded for further in silico (absorption, distribution, metabolism, excretion) ADME evaluation.

### 2.2. In Silico Pharmacokinetic Studies

The ADME properties of 1880 molecules were evaluated in this step to assess their drug-likeliness and pharmacokinetic properties. The drug-likeliness properties of the analogues were assessed by their conformation to Lipinski’s rule of five (molecular weight < 500; QPlogP_o/w_ < 5, H-B donors ≤ 5, and H-bond acceptors ≤ 10). Since the drug targets Alzheimer’s disease and would work as a BACE-1 inhibitor, the analogues were filtered for BBB permeability. Of all the analogues evaluated, only 50 molecules were selected as they possessed both drug-likeliness properties and BBB permeability (Table 2). These 50 compounds exhibited an acceptable range of physicochemical and pharmacokinetic parameters and high GI absorption. Hence, the predicted ADME properties illustrated all the sorted analogues as good drug candidates, which proceeded further for molecular docking analysis.

### 2.3. Molecular Docking

Analysis of molecular docking interactions of the selected analogues is given in Table 3. Out of the 51 molecules docked (50 analogues and elenbecestat), most of the formulated analogues showed interactions with both of the catalytic dyad residues (Asp32 and Asp228) of the BACE-1 protein via van der Waal interactions, pi-anion, halogen fluorine interactions, or carbon–hydrogen bonding. These molecules also showed significant interaction with other important amino acid residues of the BACE-1 protein like Trp115, Gly34, Gly230, Thr231, Thr232, Val332, Lys224, Arg235, and Tyr71.

**Figure 1:**
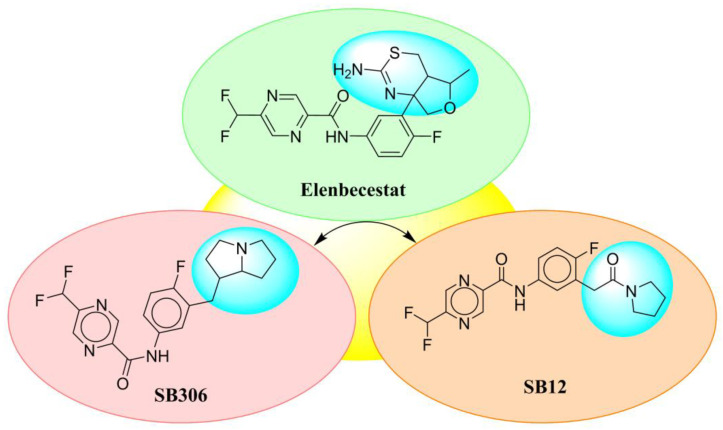
Diagrammatic representation of the best two analogs of elenbecestat with specific bioisosteric replacement as BACE-1 inhibitors for Alzheimer’s therapeutics.

Only one molecule out of all these SB282 showed no interactions with any of the catalytic dyad residues. Of all the docked molecules, the top two molecules with the maximum docking score or minimum binding energy were SB306 and SB12 (Figure 1).

**Figure 2:**
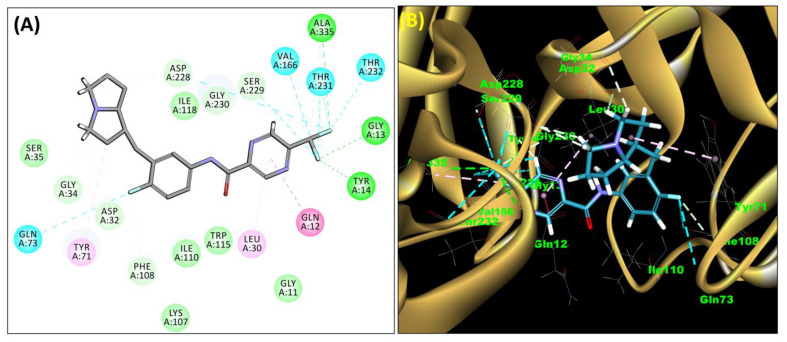
The 2D (**A**) and 3D (**B**) view showing a docked complex of SB306 with BACE-1 enzyme.

**Figure 3:**
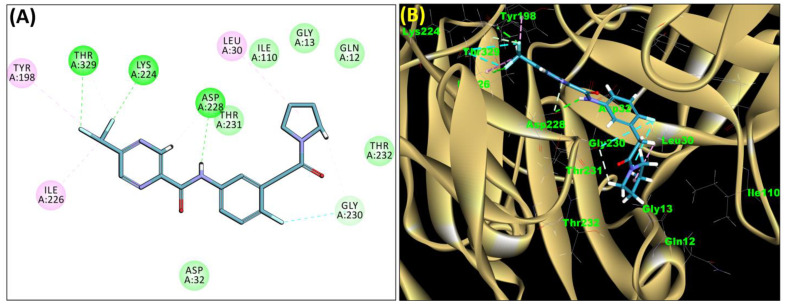
The 2D (**A**) and 3D (**B**) view showing a docked complex of SB12 with BACE-1 enzyme.

The SB306 has the most efficient binding with the BACE-1 protein with CDOCKER interaction energy (−44.3515 kcal/mol). The SB306 analogue had a Hexahydro-1*H*-pyrrolizine ring substituent in place of a 5-Methyl-4a,5,7,7a-tetrahydro-4*H*-furo [3,4-d][1,3] thiazine-2-amine ring at the fluorophenyl ring in elenbecestat, the rest of the structure was similar. The SB306 analogue showed carbon–hydrogen bond interaction with the ASP32 residue through the hydrogen of the pyrrolizine ring and van der Waals interaction with the ASP228. The fluorine atoms in the structure show maximum interactions with the amino acid residues of the binding site. The two fluorine atoms of the difluoromethyl substitution at the pyrazine ring showed halogen fluorine bonding with the THR231, SER229, and GLY230 and carbon-hydrogen bonding with the GLY13. The methyl group showed alkyl interaction with VAL166 and ALA335. The pyrazine ring showed amide-pi stacked interaction with the GLN12 and pi-alkyl interaction with LEU30. The hydrogen of the pyrazine ring shows carbon–hydrogen bonding with SER229. Another selected analogue SB12 had the second-best docking score, with CDOCKER interaction energy being −43.3585 kcal/mol. The analogue had a 2-oxo-2-pyrrolidin-1-yl ethyl ring substituent in place of the 5-Methyl-4a,5,7,7a-tetrahydro-4*H*-furo [3,4-d][1,3]thiazine-2-amine ring at the fluorophenyl ring in elenbecestat. It showed van der Waals interaction with ASP32, conventional hydrogen bonding, and carbon–hydrogen bond interaction with ASP228. The ASP228 had conventional hydrogen bonding interaction with the amide substituent of the carboxamide moiety and carbon–hydrogen bond interaction with the hydrogen of the pyrazine ring. The hydrogens of the pyrrolidine ring formed carbon–hydrogen bonding with GLY230 residue, and the two fluorine atoms of the difluoromethyl substitution showed a significant number of interactions like conventional hydrogen bonding and carbon–hydrogen bonding with the LYS224 and THR329. The methyl group showed alkyl interactions with ILE226 and TYR198. The pyrrolidine ring showed pi-alkyl interactions with LEU30. The rest of the neighboring amino acids like ILE118, SER35, ARG235, VAL332, THR231, THR232, VAL166, GLY11, GLY13, ILE110, and TRP115 showed van der Waals interactions with the molecule. The elenbecestat had the CDOCKER interaction energy of −42.8232 kcal/mol, which proves the newer analogues to be promising candidates for further research in BACE-1 inhibition. The 2D and 3D views of interactions of SB306 and SB12 are given in Figure 2 and Figure 3, respectively.

## 3. Molecular Dynamics Simulations

**Figure 4:**
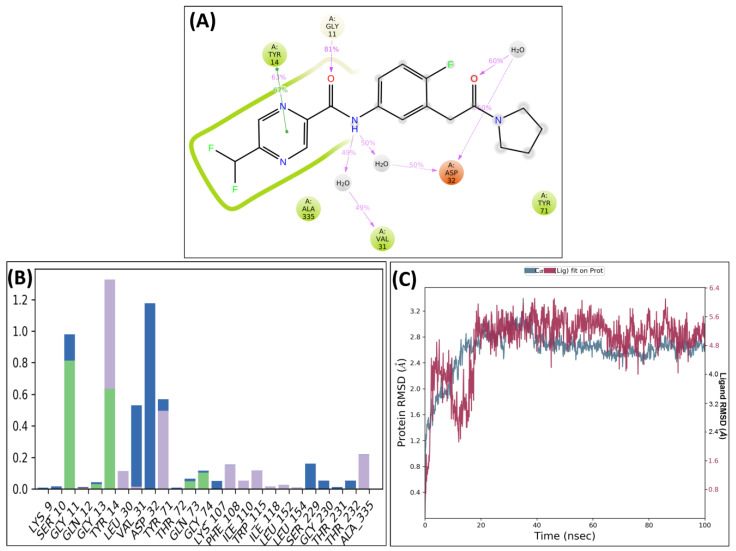
Post-MD H-bonds and hydrophobic interactions of SB306 with BACE 1. (**A**) Protein interactions fractions with the ligand (SB306) plot throughout the simulation. (**B**) Protein–ligand contacts plot of compound SB306 with BACE 1 and (**C**) RMSD trajectory plot for compound SB306.

An MD simulation study of the best two compounds was performed to analyze their thermodynamic stability and dynamic behavior of the ligand–protein complex and to study the effect on the conformational alterations induced by ligand binding with the active pocket of BACE-1 [19]. After MD, although the important interactions were conserved, some additional interactions were also observed. The protein–ligand complex of BACE-1 and SB306 were analyzed, and the compound SB306 showed hydrogen bonding interactions with the ASP32, GLY11, π-π interactions with TYR14. Strong hydrogen bonding interactions were also observed with the VAL31 (Figure 4A). These interactions are summarized in the protein–ligand contacts plot (Figure 4B). After MD simulations, the root mean square deviation (RMSD) was calculated for the SB306 compound concerning protein and plotted against time (ns) (Figure 4C).

**Figure 5:**
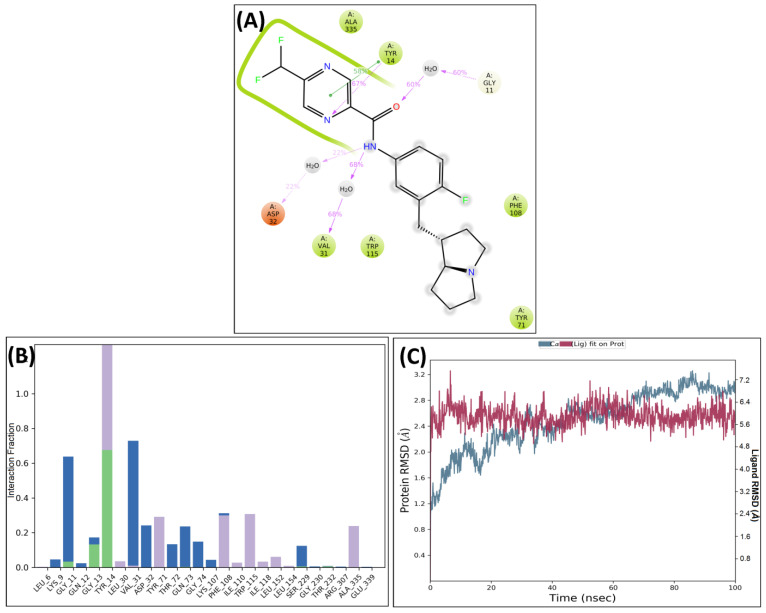
Post-MD H-bonds and hydrophobic interactions of SB12 with BACE 1. (**A**) Protein interactions fractions with the ligand (SB12) plot throughout the simulation. (**B**) Protein–ligand contacts plot of compound SB12 with BACE 1 and (**C**) RMSD trajectory plot for compound SB12.

The protein–ligand complex of BACE-1 and SB12 were also analyzed, and the compound showed strong hydrogen bonding interactions with ASP32 and VAL31. The compound showed strong π–π interactions with TYR14 and hydrogen bonding interactions with GLY11 (Figure 5A). These interactions are summarized in the protein–ligand contacts plot (Figure 5B). After the MD studies, the RMSD graph of the SB12 ligand concerning BACE-1 was plotted against time (ns) (Figure 5C).

The plots of RMSD of the compounds SB306 and SB12 showed that docked complexes were quite stable throughout, with minor fluctuations in the range of 1.2–2.0 Å. The stability of the simulated system indicates the inhibitory nature of the SB306 and SB12 on BACE 1. Compound SB306 has shown almost no or a very small vibration throughout the simulation and remains stable within the macromolecular cavity, while the ligand SB12 has shown a couple of moves during the initial 20 ns time within the binding cavity to achieve the most stable conformation. Both the compounds SB306 and SB12 have shown a high degree of stability within the macromolecular cavity throughout the simulation period, which was expected to initiate the therapeutic response.

## 4. Material and Methods

### 4.1. Clinical Trials Screening

The clinical trials database (clinicaltrials.gov.in) was screened for small molecule BACE inhibitors. A total of 7 molecules were found, listed in Table 4. Most of the molecules currently undergoing clinical trials reduced concentration of Aβ in CSF, but their trials were terminated due to liver toxicity and severe adverse effects. Elenbecestat (E2609), which is currently undergoing phase III clinical trials, was found to be effective in reducing the Aβ levels in CSF and plasma, and also resulted in a decreased rate of decline in cognitive abilities, and also no hepatic toxicity was found to occur, but some side effects are associated with the molecule [12,20].

### 4.2. Scaffold Morphing

**Figure 6:**
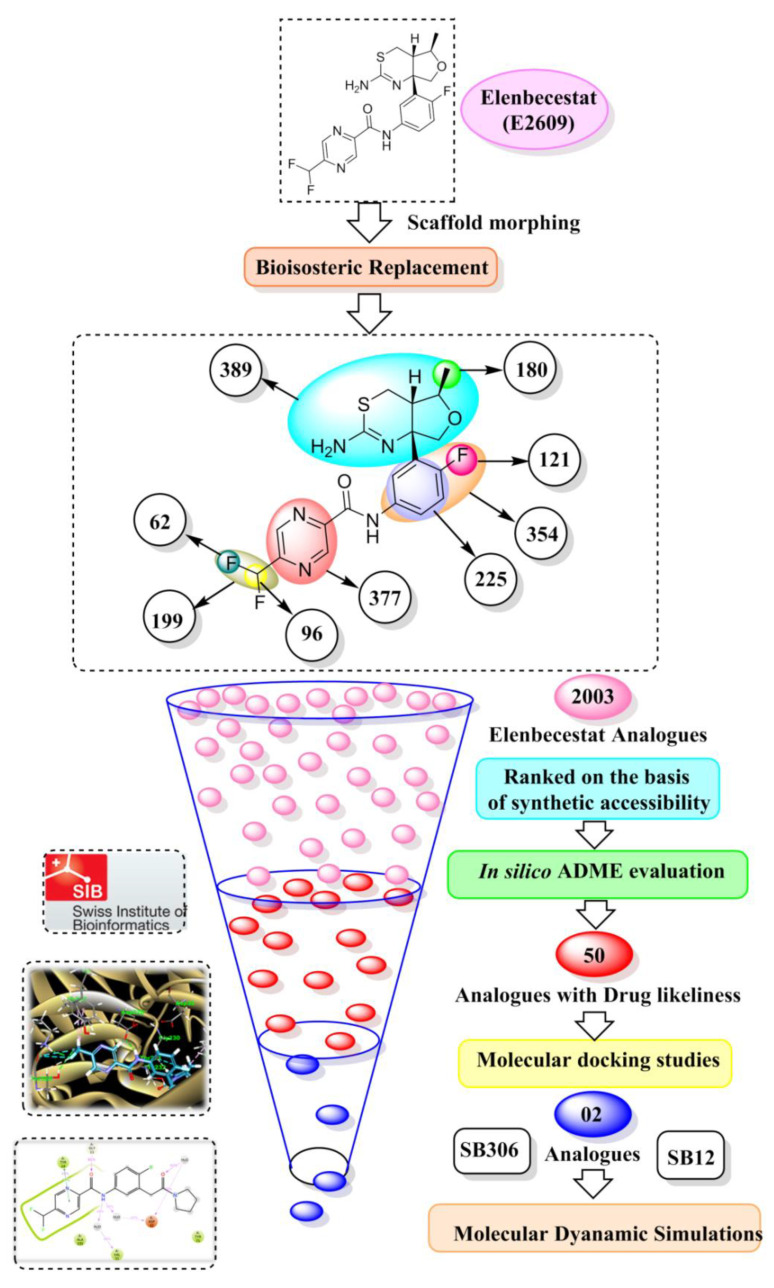
The overall workflow of the present study.

This technique for designing drugs refers to modifying structural features to ameliorate the synthetic feasibility, potency, and drug-likeliness properties. The method provides the approach to enhancing the overall therapeutic profile of the chosen molecule [21,22]. The scaffold morphing works on the principle of bio-isosteric replacement, replacing each functional group or segment of the molecule with their bioisosteres and boosting the effectiveness in addition to pharmacokinetic profiling of the compound [21,22]. In this research study, the scaffold morphing of elenbecestat was performed using the freely available web server MolOpt [18]. The three molecule sites were identified, and various analogues were generated by replacing multiple molecule segments using 4 inbuilt transformation rules: AI generative model, data mining, data mining (fast), and similarity comparison. The generated elenbecestat analogues were sorted based on synthetic accessibility. The range of synthetic accessibility lies from very easy (1) to very difficult (10) [23]. A cut-off of 4.5 was employed to screen the formed analogues, and the 1880 analogues proceeded for in silico pharmacokinetic studies (Figure 6).

### 4.3. In Silico Pharmacokinetic Predictions

The pharmacokinetic properties of the selected 1880 molecules were evaluated using the online free web server swissADME tool (http://www.swissadme.ch (accessed on 25 May 2023)). Various molecular attributes were examined, including physicochemical properties, water solubility, pharmacokinetics, lipophilicity, and drug likeliness properties comprising (Ro5) Lipinski’s rule [23,24]. Since the drug being evaluated is for Alzheimer’s disease, the BBB permeability is also considered along with other parameters for evaluating the BACE inhibitor analogues [25,26,27]. Only the analogues exhibiting BBB permeability were proceeded for further molecular docking studies. Out of 1880 molecules examined, only 50 molecules were found to possess drug-likeliness properties and BBB permeability.

### 4.4. Molecular Docking Studies

To explore the binding affinity of the formed and sorted elenbecestat analogues with the BACE-1 enzyme, docking was carried out using Biovia Discovery Studio software [24,28]. Molecular docking of the formulated analogues and elenbecestat molecule was performed using the crystal structure of BACE-1 protein possessing 2Å, and an R-value of 0.223 was obtained using (Research Collaboratory for Structural Bioinformatics) RCSB-protein data bank (PDB) (https://www.rcsb.org/ (accessed on 25 May 2023)), the PDB ID: 6OD6 [29,30,31]. The protein was prepared using the ‘macromolecule module’ of the Biovia discovery studio software. The ligands were prepared using the Discovery Studio software’s ‘small molecule module.’ The docking was proceeded by the Dock ligands ‘CDOCKER’ protocol, and the docked poses’ molecular interactions was noted down [19]. In docking experiments, the grid box was generated with the X, Y, and Z axes coordinates of −36.937, −45.388, and 19.516, respectively, for 6OD6. A maximum of 10 binding modes were allowed to be generated for each ligand during the docking execution program [32,33,34]. Before docking the ligands, the docking protocol was validated by redocking the co-crystallized ligand (ligand ID: M7D) attached to the BACE-1 protein. Similar validated docking protocols for the analysis of the prepared ligand were followed. The interactions of the docked ligands with the catalytic aspartate (Asp) dyad (Asp32 and Asp228) residues present in the BACE-1 protein were observed. The interactions, docking score, and binding energy for each docked ligand were noted.

### 4.5. Molecular Dynamic Simulations

The designed analogues having good binding energies against the BACE-1 enzyme in molecular docking were further assessed for their thermodynamic stability concerning time by executing molecular dynamics (MD) simulation for a duration of 100 ns by using the molecular dynamics module of Desmond software by Schrodinger [35,36,37,38]. The simulations aided in stabilizing the protein–ligand complex by minimizing the system’s energy by attaining the most preferred conformation after complexation concerning time. The interaction persisting between the complex ligand and the target macromolecule during the 100 ns duration of the simulation was analyzed by studying its simulation–interaction diagram [37,39,40,41,42,43,44]. This analysis leads to assessing important interactions for ligand binding that can be utilized to assess ligand’s affinity further. The system was first built using the TIP3P solvent model with an orthorhombic box shape; the input system’s ionic state was adjusted using the salt solution of 0.15 M. The simulation was carried out using the NPT ensemble and a time step of 1.0 fs; the constant temperature of 310 K was adjusted by using the Nose–Hoover Chain method as the thermostat and pressure of 1.01325 bar using Martyn–Tobias–Klein as the barostat.

## 5. Conclusions and Future Remarks

A scaffold morphing and a structure-based drug designing approach were successfully utilized to identify putative analogues of elenbecestat against BACE 1. Initially, the bio-isosteric replacement was carried out on various suggested sites of elenbecestat to generate a library of its analogues. From a library of more than 10,000 analogues, 2003 were selected based on synthetic feasibility and submitted for in silico ADME prediction to confer the drug-like properties of these analogues. Using a molecular docking approach, the 50 analogues were further evaluated for binding affinity with the target protein. Though all the compounds showed good interactions with the target protein (BACE 1), compounds SB306 and SB12 were the most active molecules, suggesting a plausible binding mode with the active or catalytic site of the enzyme, which is responsible for the BACE 1 activity. To analyze the stability and dynamic behavior of the ligands–protein complexes and to study the effect on the conformational alterations induced by binding of the ligand with the active pocket of BACE 1, the molecular dynamic simulation study of the top two compounds (SB306 and SB12) was performed. The plots of RMSD of both the compounds showed stability of the complexes all through with minor fluctuations in the range of 1.2–2.0 Å. The stability of the simulated system indicated the inhibitory nature of the SB306 and SB12 on BACE 1. Based on these results, it can be suggested that a slight structural modification in the elenbecestat may improve its therapeutic profile. The protocol adopted in this study may be used as a framework in the future for developing novel small molecules for the treatment of Alzheimer’s disease and suggested that the designed new compounds could be further investigated for pharmacological development in AD therapy.

## Figures and Tables

**Table 1:** Structures of designed top 50 analogues.

S. No.	Compound ID	Structure	S. No.	Compound ID	Structure
1.	SB6	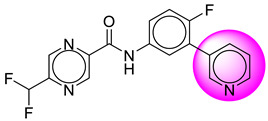	26.	SB213	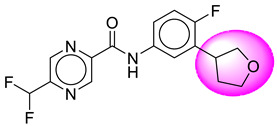
2.	SB12	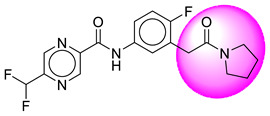	27.	SB220	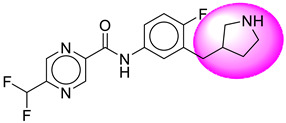
3.	SB19	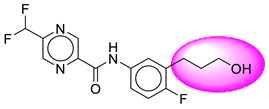	28.	SB234	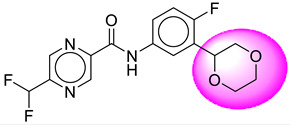
4.	SB20	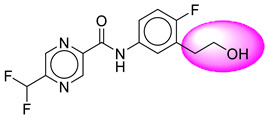	29.	SB242	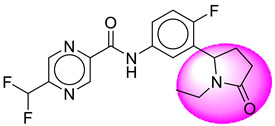
5.	SB21	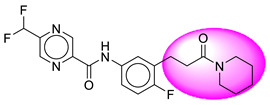	30.	SB247	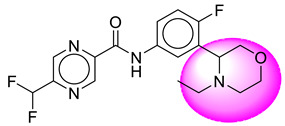
6.	SB22	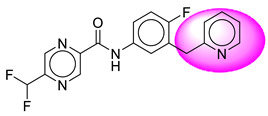	31.	SB262	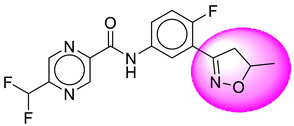
7.	SB35	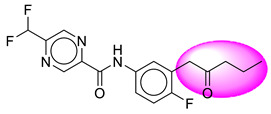	32.	SB282	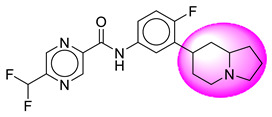
8.	SB37	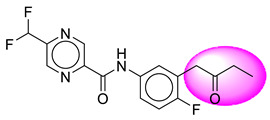	33.	SB283	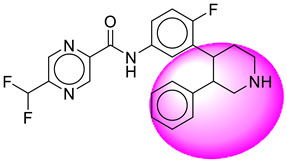
9.	SB38	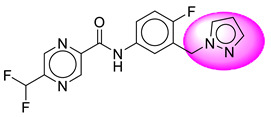	34.	SB306	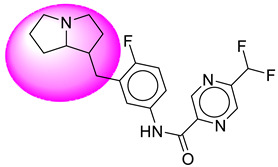
10.	SB41	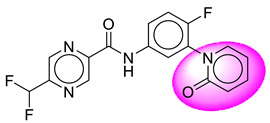	35.	SB335	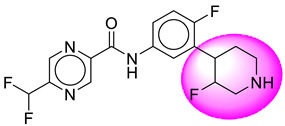
11.	SB42	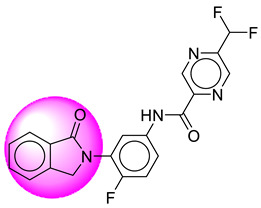	36.	SB339	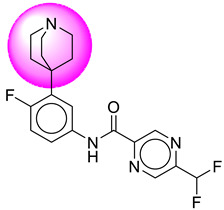
12.	SB63	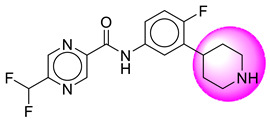	37.	SB340	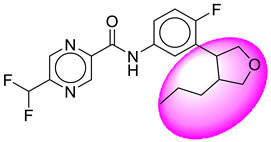
13.	SB92	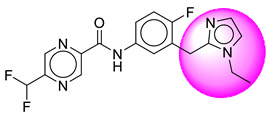	38.	SB342	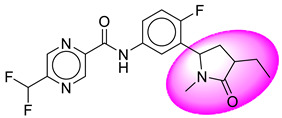
14.	SB139	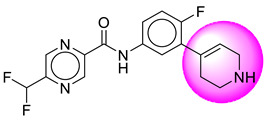	39.	SB353	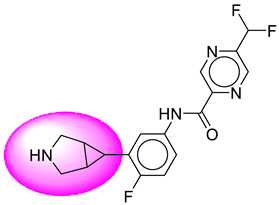
15.	SB153	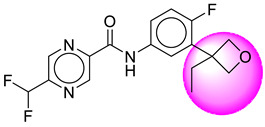	40.	SB357	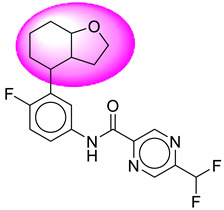
16.	SB171	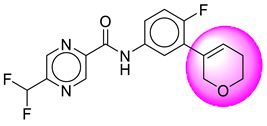	41.	SB362	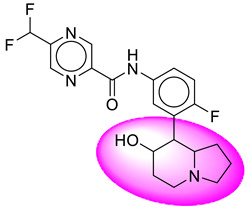
17.	SB195	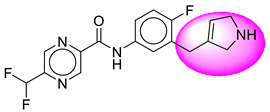	42.	SB363	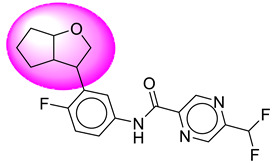
18.	SB196	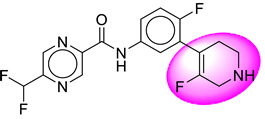	43.	SB364	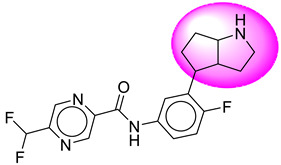
19.	SB203	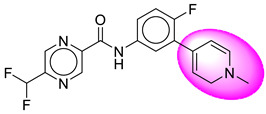	44.	SB367	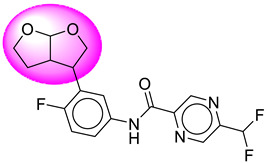
20.	SB204	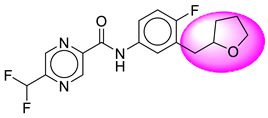	45.	SB375	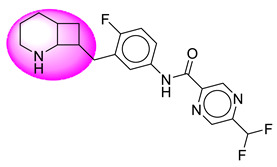
21.	SB205	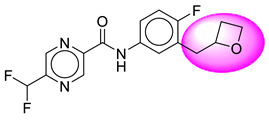	46.	SB381	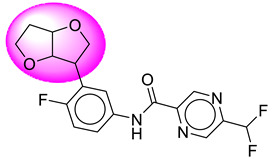
22.	SB206	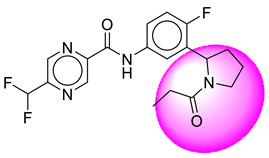	47.	SB382	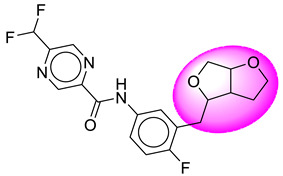
23.	SB208	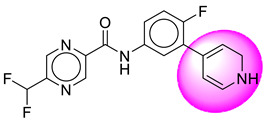	48.	SB395	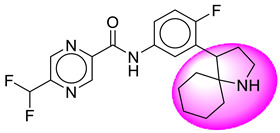
24.	SB209	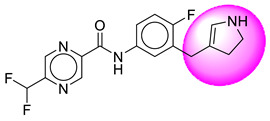	49.	SB523	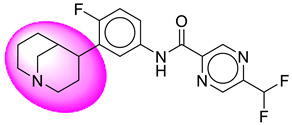
25.	SB210	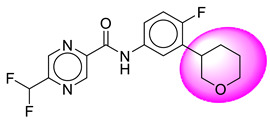	50.	SB856	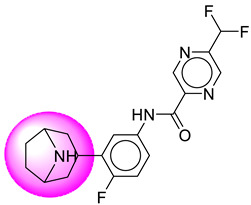

**Table 2:** Predicted ADME properties of designed top 50 analogues.

S. No.	Compound ID	MW	HBA	HBD	TPSA (Å)	Consensus Log P	Ali Log S	Lipinski Violations	BBB Permeant	GI Absorption
1	**[SB6]**	344.29	7	1	67.77	2.85	−3.32	0	Yes	High
2	**[SB12]**	378.35	7	1	75.19	2.47	−2.78	0	Yes	High
3	**[SB19]**	325.29	7	2	75.11	2.37	−2.83	0	Yes	High
4	**[SB20]**	311.26	7	2	75.11	2.01	−2.46	0	Yes	High
5	**[SB21]**	406.4	7	1	75.19	3	−3.45	0	Yes	High
6	**[SB22]**	358.32	7	1	67.77	3.09	−3.66	0	Yes	High
7	**[SB35]**	351.32	7	1	71.95	2.99	−3.29	0	Yes	High
8	**[SB37]**	337.3	7	1	71.95	2.71	−2.93	0	Yes	High
9	**[SB38]**	347.29	7	1	72.7	2.35	−2.75	0	Yes	High
10	**[SB41]**	360.29	7	1	76.88	2.44	−2.96	0	Yes	High
11	**[SB42]**	398.34	7	1	75.19	2.82	−3.52	0	Yes	High
12	**[SB63]**	350.34	7	2	66.91	2.57	−2.84	0	Yes	High
13	**[SB92]**	375.35	7	1	72.7	2.73	−3.22	0	Yes	High
14	**[SB139]**	348.32	7	2	66.91	2.47	−2.46	0	Yes	High
15	**[SB153]**	351.32	7	1	64.11	3	−3.27	0	Yes	High
16	**[SB171]**	349.31	7	1	64.11	2.81	−2.69	0	Yes	High
17	**[SB195]**	348.32	7	2	66.91	2.45	−2.28	0	Yes	High
18	**[SB196]**	366.31	8	2	66.91	2.66	−2.27	0	Yes	High
19	**[SB203]**	360.33	6	1	58.12	2.67	−3.1	0	Yes	High
20	**[SB204]**	351.32	7	1	64.11	2.99	−3.36	0	Yes	High
21	**[SB205]**	337.3	7	1	64.11	2.7	−2.99	0	Yes	High
22	**[SB206]**	392.37	7	1	75.19	2.72	−3.21	0	Yes	High
23	**[SB208]**	346.31	6	2	66.91	2.54	−3.14	0	Yes	High
24	**[SB209]**	348.32	6	2	66.91	2.63	−3.1	0	Yes	High
25	**[SB210]**	351.32	7	1	64.11	3.08	−3.08	0	Yes	High
26	**[SB213]**	337.3	7	1	64.11	2.71	−2.7	0	Yes	High
27	**[SB220]**	350.34	7	2	66.91	2.59	−2.96	0	Yes	High
28	**[SB234]**	353.3	8	1	73.34	2.08	−2.15	0	Yes	High
29	**[SB242]**	378.35	7	1	75.19	2.39	−2.66	0	Yes	High
30	**[SB247]**	380.36	8	1	67.35	2.34	−2.59	0	Yes	High
31	**[SB262]**	350.3	8	1	76.47	2.6	−3.23	0	Yes	High
32	**[SB282]**	390.4	7	1	58.12	3.28	−3.72	0	Yes	High
33	**[SB283]**	426.43	7	2	66.91	3.73	−4.29	0	Yes	High
34	**[SB306]**	390.4	7	1	58.12	3.26	−3.83	0	Yes	High
35	**[SB335]**	368.33	8	2	66.91	2.72	−2.9	0	Yes	High
36	**[SB339]**	376.38	7	1	58.12	2.95	−3.28	0	Yes	High
37	**[SB340]**	379.38	7	1	64.11	3.53	−4.09	0	Yes	High
38	**[SB342]**	392.37	7	1	75.19	2.68	−3.24	0	Yes	High
39	**[SB353]**	348.32	7	2	66.91	2.3	−2.4	0	Yes	High
40	**[SB357]**	391.39	7	1	64.11	3.56	−4.11	0	Yes	High
41	**[SB362]**	406.4	8	2	78.35	2.62	−3.13	0	Yes	High
42	**[SB363]**	377.36	7	1	64.11	3.41	−3.54	0	Yes	High
43	**[SB364]**	376.38	7	2	66.91	2.93	−3.31	0	Yes	High
44	**[SB367]**	379.33	8	1	73.34	2.55	−2.95	0	Yes	High
45	**[SB375]**	390.4	7	2	66.91	3.24	−3.8	0	Yes	High
46	**[SB381]**	379.33	8	1	73.34	2.61	−2.65	0	Yes	High
47	**[SB382]**	393.36	8	1	73.34	2.78	−3.14	0	Yes	High
48	**[SB395]**	404.43	7	2	66.91	3.62	−4.16	0	Yes	High
49	**[SB523]**	390.4	7	1	58.12	3.26	−3.54	0	Yes	High
50	**[SB856]**	376.38	7	2	66.91	3.01	−3.49	0	Yes	High

**Table 3:** Docking score and key interactions of designed elenbecestat analogs with BACE-1.

S. No.	Compound ID	Docking Score (kcal/mol)	Interactions
1	**Elenbecestat**	−42.82	Asp32, Asp228, Ser35, Ile 118, Leu30, Gly13, Tyr14, Gly34, Ser229, Thr232
2	**SB6**	−33.54	Asp32, Tyr71, Gly230, Val166, Trp115, Thr231, Thr232, Arg235
3	**SB12**	−43.35	Asp32, Asp228, Thr329, Arg235, Gly230, Thr232, Val166, Thr231, Trp115, Gly34, Lys224, Thr329, Tyr198, Val332, Leu30, Ile226
4	**SB19**	−37.70	Asp32, Asp228, Thr232, Arg235, Gly34, Gly230, Thr231, Val166,
5	**SB20**	−34.53	Asp32, Asp228, Gly34, Gly230, Thr231, Arg235, Thr232, Val166,
6	**SB21**	−43.06	Asp32, Asp228, Arg235, Val332, Gly230, Gly34, Thr329, Lys224, Thr231, Val166, Thr232
7	**SB22**	−38.46	Asp32, Asp228, Gly34, Tyr71, Trp115, Gly230, Thr231, Thr232, Val166
8	**SB35**	−41.27	Asp32, Asp228, Thr232, Thr231, Gly230, Val332, Arg235, Gly34, Trp115, Val166
9	**SB37**	−38.47	Asp32, Asp228, Gly34, Arg235, Thr232, Thr231, Gly230, Val166
10	**SB38**	−38.04	Asp32, Asp228, Val166, Gly34, Tyr71, Trp115, Gly230, Thr231, Thr232
11	**SB41**	−37.06	Asp32, Val166, Thr232, Thr231, Tyr71
12	**SB42**	−35.51	Asp32, Asp228, Tyr71, Gly34, Gly230, Lys224, Val332, Trp115, Val166, Thr329, Thr231, Thr232
13	**SB63**	−41.42	Asp32, Asp228, Gly34, Trp115, Gly230, Val166, Thr231, Thr232
14	**SB92**	−43.31	Asp32, Asp228, Gly34, Arg235, Thr232, Thr231, Gly230, Val166, Trp115
15	**SB139**	−36.55	Asp32, Asp228, Tyr71, Gly34, Gly230, Val332, Trp115, Val166, Thr231, Thr232
16	**SB153**	−32.568	Asp32, Asp228, Gly230, Thr231, Thr232, Trp115, Tyr71, Val332, Lys224, Thr329, Arg235
17	**SB171**	−33.11	Asp32, Asp228, Trp115, Tyr71, Thr231, Thr232, Gly230, Gly34
18	**SB195**	−41.40	Asp32, Asp228, Gly34, Gly230, Thr231, Arg235, Thr232, Val166, Trp115, Tyr71
19	**SB196**	−37.88	Asp32, Asp228, Gly34, Trp115, Gly230, Val166, Thr231, Thr232
20	**SB203**	−38.86	Asp32, Gly34, Tyr71, Trp115, Gly230, Thr231, Thr232, Val166
21	**SB204**	−42.01	Asp32, Asp228, Gly34, Gly230, Thr231, Thr232, Val166, Trp115
22	**SB205**	−34.14	Asp32, Asp228, Gly34, Val332, Thr231, Thr232, Gly230, Trp115, Tyr71, Val166
23	**SB206**	−37.43	Asp32, Asp228, Gly34, Gly230, Thr231, Thr232, Thr329, Trp115, Lys224, Val332, Arg235
24	**SB208**	−30.56	Asp32, Asp228, Gly34, Gly230, Thr231, Tyr71
25	**SB209**	−31.31	Asp32, Asp228, Tyr71, Gly34, Trp115, Gly230, Val166, Thr232, Thr231
26	**SB210**	−40.6	Asp32, Trp115, Thr231, Thr232, Val166, Gly34, Tyr71, Gly230
27	**SB213**	−33.89	Asp32, Asp228, Tyr71, Gly34, Val332, Thr231, Thr232, Gly230, Trp115
28	**SB220**	−40.46	Asp32, Asp228, Gly34, Gly230, Tyr71, Trp115, Val166, Thr232, Thr231
29	**SB234**	−33.02	Asp32, Asp228, Tyr71, Gly34, Gly230, Thr231, Thr232, Trp115
30	**SB242**	−37.75	Asp32, Arg235, Tyr71, Gly230, Thr231, Thr232, Val166, Trp115
31	**SB247**	−36.85	Asp228, Gly230, Val166, Trp115, Thr231, Thr232, Thr329, Arg235, Val332
32	**SB262**	−40.15	Asp32, Trp115, Thr231, Thr232, Val166, Gly34, Tyr71, Gly230
33	**SB283**	−43.30	Asp32, Asp228, Trp115, Tyr71, Thr231, Thr232, Gly230, Gly34, Val166
34	**SB306**	−44.35	Asp32, Asp228, Gly34, Tyr71, Trp115, Gly230, Thr231, Thr232, Val166
35	**SB335**	−33.29	Asp32, Asp228, Gly34, Gly230, Val332, Arg235, Lys224, Thr329, Thr231, Thr232, Trp115, Tyr71
36	**SB339**	−41.84	Asp32, Asp228, Gly34, Tyr71, Trp115, Gly230, Val166, Thr231, Thr232
37	**SB340**	−39.28	Asp32, Asp228, Tyr71, Gly34, Gly230, Thr231, Thr232, Trp115, Val166
38	**SB342**	−38.96	Asp32, Asp228, Val166, Gly34, Tyr71, Trp115, Gly230, Thr231, Thr232, Arg235, Thr329 Lys224, Val332
39	**SB353**	−39.54	Asp32, Asp228, Trp115, Gly34, Gly230, Tyr71, Val166, Thr231, Thr232
40	**SB357**	−39.79	Asp32, Asp228, Val166, Gly34, Gly230, Tyr71, Trp115
41	**SB362**	−41.43	Asp32, Asp228, Thr329, Thr231, Thr232, Lys224, Val332, Gly34, Gly230, Tyr71
42	**SB363**	−42.46	Asp32, Asp228, Val332, Thr231, Thr232, Thr329, Lys224, Gly230, Gly34, Tyr71
43	**SB364**	−35.77	Asp32, Asp228, Thr231, Thr232, Gly34, Gly230, Val332, Tyr71, Thr329, Lys224
44	**SB367**	−41.62	Asp32, Asp228, Gly34, Tyr71, Thr231, Gly230, Thr232, Val332, Lys224, Thr329
45	**SB375**	−38.84	Asp32, Asp228, Gly34, Gly230, Thr231, Thr329, Tyr71, Val332, Lys224
46	**SB381**	−41.17	Asp32, Asp228, Gly34, Gly230, Tyr71, Thr231, Val332, Thr329, Lys224
47	**SB382**	−39.06	Asp32, Asp228, Lys224, Thr329, Val332, Gly34, Gly230, Thr231, Tyr71
48	**SB395**	−33.28	Asp32, Asp228, Val332, Gly230, Gly34, Thr231, Tyr71, Thr329, Lys224
49	**SB523**	−40.50	Asp32, Asp228, Gly34, Gly230, Lys224, Thr329, Val332, Thr231, Tyr71
50	**SB856**	−40.44	Asp32, Asp228, Lys224, Thr329, Val332, Gly34, Gly230, Thr231, Tyr71

**Table 4:** Some of the small molecule inhibitors are under clinical trials.

Molecule Name	Clinical and Pharmacology Consideration	Clinical Trial Status	Clinical Trial ID
Lanabecestat (AZD3293, LY3314814)	Reduce CSF Aβ levels	Phase 3 trials terminated due to less efficacy	Clinicaltrials.gov ID: NCT02245737
(JNJ-54861911) Atabecestat	Reduce CSF Amyloid-β levels	Phase 2 or 3 trials were terminated due to liver toxicity	Clinicaltrials.gov ID: NCT02569398
CNP520	Reduce CSF Aβ levels	Phase II/III completed	Clinicaltrials.gov ID: NCT02576639
(E2609) Elenbecestat	Decreased Amyloid-β levels in cerebrospinal fluid and plasma Less decline in functional cognition.	Currently in Phase III clinical trials but side effects like dermatitis, respiratory tract infection, abnormal nightmares and dreams, headache, falls, and diarrhea but safe for liver	Clinicaltrials.gov ID: NCT03036280, NCT02956486
(MK-8931) Verubecestat	Decreased CNS amyloid-β levels in animals and patients of AD	Phase 3 trials were withdrawn due to lack of efficacy as well as dermatological and behavioral side effects	Clinicaltrials.gov ID: NCT01953601
LY2886721	Potent BACE-1 inhibitor	Phase 1/2 terminated due to abnormal liver biochemical toxicity.	Clinicaltrials.gov ID: NCT01561430
LY320262	BACE-1 inhibitor	Phase II but no clinical efficacy as of now	Clinicaltrials.gov ID: NCT02323334

## Data Availability

The data presented in this study are available within the article.
